# Elucidating the role of APOE ε4 gene variants in the clinical manifestation of Parkinson's disease

**DOI:** 10.3389/fnagi.2025.1632480

**Published:** 2025-10-17

**Authors:** Gita Vita Soraya, Yusran Ady Fitrah, Andi Kurnia Bintang, Muhammad Akbar, Alfi Raudatil Jannah, Zulvikar Syambani Ulhaq, Jumraini Tammasse, Cahyono Kaelan, Muhammad Nasrum Massi, Ai Obinata, Norikazu Hara, Akinori Miyashita, Takeshi Ikeuchi

**Affiliations:** ^1^Department of Biochemistry, Faculty of Medicine, Hasanuddin University, Makassar, Indonesia; ^2^Department of Neurology, Faculty of Medicine, Hasanuddin University, Makassar, Indonesia; ^3^Department of Biomedicine and Molecular Biology, School of Postgraduate Studies, Hasanuddin University, Makassar, Indonesia; ^4^Wahidin Sudirohusodo General Hospital, Makassar, Indonesia; ^5^Department of Molecular Genetics, Brain Research Institute, Niigata University, Niigata, Japan; ^6^Research Center for Preclinical and Clinical Medicine, National Research and Innovation Agency, Cibinong, Indonesia; ^7^Department of Microbiology, Faculty of Medicine, Hasanuddin University, Makassar, Indonesia

**Keywords:** Parkinson's disease, apolipoprotein gene, movement disorder, onset age, severity

## Abstract

**Introduction:**

Parkinson's Disease (PD) is the most common movement disorder, and remains a major cause of mortality and morbidity worldwide. Studies have uncovered the potential role of the Apolipoprotein (APOE) gene in PD, although results have been conflicting. This study aimed to characterize the APOE ε4 status of Indonesian PD subjects and perform a meta-analysis to elucidate it's role in PD onset age, disease severity, and cognition.

**Methods:**

APOE ε4 genotyping was performed on PD patients and their respective healthy families. Clinical parameters were obtained from PD patients. Secondly, we conducted a meta-analysis on the role of APOE ε4, with studies collected until January 2025. Retrieved parameters include the number of PD APOE ε4 allele carriers and non-carriers, age of onset, disease severity, and mini-mental state examination (MMSE) scores.

**Results:**

Four of the 7 recruited subjects were APOE ε4 carriers, with 2 out of 3 PD subjects of APOE ε4 carrier status. In the meta-analysis, 14 included studies revealed a significantly younger age of onset in APOE ε4 carriers (SMD = −0.16, 95%CI −0.24 to −0.08, *p* = 0.0001) relative to non-carriers. Six included studies revealed no significant difference in the Hoehn and Yahr disease severity, and 4 included studies showed no significant difference in MMSE scores of carriers vs. non-carriers.

**Conclusion:**

The APOE ε4 allele is common in this preliminary study of 3 PD subjects. Our meta-analysis revealed a significantly earlier age-of-onset among APOE ε4 carriers relative to non-carriers, but no difference in disease severity and MMSE scores.

## Introduction

To date, approximately 2%−3% of the population aged ≥65 years suffers from Parkinson's Disease (PD) (Poewe et al., 2017), a movement disorder that remains a significant burden worldwide. This is not only due to its increasing prevalence in the aging population but also due to its toll on global disability and mortality rates (Dorsey et al., 2018). The burden is largely due to the presence of debilitating motor and non-motor symptoms, manifesting in a range of issues such as resting tremors, rigidity, bradykinesia, gait abnormalities, postural instabilities, autonomic complaints, and cognitive dysfunction (Bergman and Deuschl, 2002; Lindenbach and Bishop, 2013).

Pathologically, PD is marked by the accumulation of α-synuclein protein and -Lewy body formation, which overall leads to the degeneration of dopaminergic neurons, particularly within the substansia nigra (Moore et al., 2005; Schulz-Schaeffer, 2010). Multiple efforts over recent years have aimed to characterize the role of genetics on the risk of PD development (Alcalay et al., 2020), progression, severity, and therapeutic response (Soraya et al., 2022). One particular gene of interest is Apolipoprotein E (APOE), particularly APOE ε4 allele status. Classically, the APOE ε4 allele is known as one of the most common genetic determinants of Alzheimer's Disease (AD) (Sullivan et al., 2011). Due to its multiple potential mechanisms in AD pathology portrayed through both cellular and animal models, it has even been proposed as a therapeutic target in AD (Safieh et al., 2019). While the role of APOE ε4 has been well established in AD, previous studies have been conflicting regarding the potential role of APOE ε4 in PD susceptibility risk and onset age (Li et al., 2004; Federoff et al., 2012), disease severity and progression (Jo et al., 2021), as well as the extent of cognitive impairment and rate of cognitive decline (Aarsland et al., 2017; Fagan and Pihlstrøm, 2017) especially in the context of earlier development of PD Dementia (Pang et al., 2018).

Despite growing interest in the role of APOE ε4 in PD, the gene is rarely characterized in Indonesian populations. Previous genetic studies on the healthy Indonesian population have shown a distinct APOE distribution across the nation, wherein a higher frequency of APOE ε3 and ε4 were found in the Eastern part of Indonesia, relative to the western and middle regions (Hastuti et al., 2015). Due to the implications of APOE in several cardiac and cognitive pathomechanisms, many studies have focused on its associations with conditions such as AD, or heart diseases. However, no studies have characterized the APOE gene in the Indonesian PD population.

In part one of this study, we aimed to perform APOE characterization in 3 families of PD patients of Eastern Indonesian background (South and West Sulawesi). In the second part of this study, we aimed to clarify the role of APOE ε4 on the onset, clinical severity, and cognition of PD patients through a meta-analysis approach.

## Materials and methods

### Study population and clinical assessment

All subjects were recruited from the Wahidin Sudirohusodo General Hospital in Makassar, Indonesia. Ethical approval (Hasanuddin University Ethics Number: 248/UN4.6.4.5.31/PP36/2024, Niigata University Ethics Number: G2024-0020) and written informed consent from patients and respective family members were obtained before study commencement. Parkinsons Disease subjects were recruited based on a PD diagnosis in accordance with the UK Parkinson's Disease Society Brain Bank Diagnostic Criteria, in addition to family history of PD. The disease severity was determined using the Hoehn and Yahr scale and the Movement Disorder Society – Unified Parkinson's Disease Rating Scale (MDS-UPDRS). Cognitive assessment was conducted on PD subjects using the Mini-Mental State Exam (MMSE).

### APOE ε4 genotyping

We obtained a sample of whole blood and performed subsequent DNA extraction (Geneaid Biotech Ltd) on the patients (and additional healthy family members), at the Hasanuddin University Medical Research Centre (HUMRC) laboratory, Indonesia. This was followed by APOE genotyping targeting rs429358 and rs7412 at the Brain Research Institute, Niigata University, Japan. The APOE gene was amplified by a polymerase chain reaction (PCR) using the following forward (5′-GCGTACAAATGGGAACCTGGA-3′) and reverse (5′-ACGGTGCTGTCCATCTCCTG-3′) primers (Thermo Fisher Scientific Inc), thereby producing a 491 bp fragment. The PCR reaction mixture (10 μL) was composed of 1.0 μL genomic DNA (7–10 ng/μL), 5.0 μL 2 × GC buffer II (TaKaRa), 1.0 μL 2.5 mM dNTPs, 1.0 μL primer mixture (5 μM each), 0.1 μL LA Taq polymerase (5 U/μL), and 1.9 μL distilled water. The PCR parameters were 5 min of 93 °C denaturation, 33 cycles of 1 min at 93 °C denaturation, 1 min at 60 °C annealing, and 2 min at 72 °C extension. A final extension step was performed for 7 min at 72 °C, and samples were then stored at 15 °C.

Following the PCR, products were decontaminated with Exo SAP-IT (GE Healthcare) to strip off residual primers and dNTPs. Three volumes of diluted buffer 10 × PCR were prepared by diluting Exo SAP-IT for immediate use. The cleanup reaction of 14 μL included 10.0 μL PCR product and 4.0 μL solution of diluted Exo SAP-IT, which was then incubated at 37 °C for 20 min, followed by enzyme inactivation by heating to 80 °C for 15 min. These purified products were further characterized with an ABI 3130 xl Genetic Analyzer (Applied Biosystems, Thermo Fisher Scientific) for sequencing. Genotyping was performed by detecting single nucleotide polymorphisms (SNPs) at rs429358 and rs7412 to determine the APOE alleles (^*^ε2^*^, ^*^ε3^*^, ^*^ε4^*^). Quality control was performed by these three methods: (1) assessment of chromatograms for clear, sharp peaks; (2) assessing base-calling accuracy with Phred quality scores, and (3) confirmation of alignment with a reference sequence. Furthermore, APOE genotyping was also carried out using the TaqMan assay, followed by confirmation of consistency with Sanger sequening results. All Sanger Sequencing was performed in a blinded manner, with anonymized sample identifiers.

### Meta analysis

The meta-analysis was performed according to the Preferred Reporting Items for Systematic Reviews and Meta-Analysis (PRISMA) guidelines (Page et al., 2021). To obtain the dataset for the meta-analysis, we first performed a database search by querying two databases (PubMed and ScienceDirect) using the search terms “Parkinson's Disease” in combination with the terms “APOE” or “apolipoprotein E.” The search was performed with a cutoff of January 2025, with no date, location, or language restrictions. Studies were included on the basis of the following criteria: (1) articles written in English, (2) conducted on PD patients diagnosed using either the Hughes, UK-PDS or Holler criteria, and (3) reported the APOE ε4 genotype (4) reported relevant clinical information including either age of onset, disease severity (measured using the Hoehn and Yahr or MDS-UPDRS), and global cognition (measured using the MMSE).

The article selection process was performed by two independent reviewers (G.V.S and Z.S.U), and disagreements were resolved through discussion, and if necessary, resolved by a third reviewer (Y.A.F). The data collected included: (1) the name of the first or primary author, (2) publication year, (3) the country where the study originated or the ethnicity of the study population, (4) the age of onset or the age at which PD symptoms began, (5) the number of subjects with and without the APOE ε4 genotype, (6) the PD severity scores, and (7) global or overall cognitive performance scores. If any missing data was encountered, then the study is excluded. The values extracted represented the mean and standard deviation for each of these parameters.

Prior to performing summary measures, all studies were subject to quality analysis using the Newcastle-Ottawa Scale by the reviewers, and all studies of moderate to high quality (scores of 5–9) were included in the analysis. To analyze differences in measurements between individuals with and without the APOE ε4 genotype, we calculated the pooled standardized mean difference (SMD) along with a 95% confidence interval (CI). The heterogeneity among studies was assessed using the Q test and the I^2^ statistic. A significant Q statistic (p <0.10) indicated the presence of heterogeneity across the studies. I^2^ values were interpreted as indicating no (0%−24.9%), low (25%−49.9%), moderate (50%−74.9%), or high (75%−100%) heterogeneity. A random-effects model (REM) was applied for all analyses. Meta-analysis was conducted using RevMan version 5.4.1. Publication bias was assessed using the Egger's test and funnel plot generation using R software (version 4.3.1; R Foundation for Statistical Computing, Vienna, Austria) with the metafor package (https://rdrr.io/cran/metafor/). Statistical testing were two-tailed and a significance threshold set at *p* < 0.05.

## Results

### APOE ε4 genotyping in Indonesian Parkinson's disease patients

Results of the clinical and genetic assessments are depicted in Table 1. A total of 3 PD subjects and respective family member were recruited. In the first family, the PD patient (subject 3) was a 72 year old male with a 5 year history of PD diagnosis of moderate severity, and is on routine levodopa therapy. Probands (Subjects 1–2) were healthy sons and daughters of Subject 3. In the second family, the PD patient (Subject 5) is a 71 year old female with a 6 year diagnosis of PD and is on routine Levodopa treatment. The patient was tested alongside her daughter (Subject 4) who was a healthy female. The two family members were both heterozygous ε4 genotypes. In the third family, the PD patient (Subject 6) is a 67 year old female with a 7 year diagnosis of PD and is on routine Levodopa and Arkine treatment. The patient was tested with her daughter (Subject 7) who was a healthy female. Results of APOE genotyping (Table 1) revealed that 2 PD patients of APOE ε4 carrier status and 1 non-carrier. Overall, 57% of the subjects recruited for APOE genotyping were APOE ε4 carriers.

**Table 1:** Clinical and genetic assessment of Indonesian subjects.

**Family**	**Subject**	**Clinical diagnosis**	**Gender**	**Cognition**	**MDS-UPDRS I**	**MDS-UPDRS II**	**MDS-UPDRS III**	**APOE genotype**		
								**rs429358**	**rs7412**	**Genotype**
1	1	Healthy subject	Male	Normal cognition	N/A	N/A	N/A	T/T	C/C	3^*^3
	2	Healthy subject	Female	Normal cognition	N/A	N/A	N/A	T/T	C/C	3^*^3
	3	PD	Male	Mild cognitive impairment	13	16	33	T/T	C/T	2^*^3
2	4	Healthy subject	Female	Normal cognition	N/A	N/A	N/A	T/C	C/T	2^*^4
	5	PD	Female	Mild cognitive impairment	6	10	24	T/C	C/C	3^*^4
3	6	PD	Female	Mild cognitive impairment	9	18	37	T/C	C/T	2^*^4
	7	Healthy subject	Female	Normal cognition	N/A	N/A	N/A	C/C	C/C	4^*^4

PD, Parkinsons Disease; MDS-UPDRS, Movement Disorder Society – Unified Parkinson's Disease Rating Scale; APOE, Apolipoprotein E.

### Effect of APOE ε4 carrier status on age of onset, severity, and cognition in PD

A total of 2,203 articles were identified through the electronic database search. After removal of 148 duplicates, abstract and title screening was performed on 2,055 records, yielding 52 records for full-text eligibility assessment. After the assessment, a total of 18 studies were included in the analysis, with the remaining being excluded for lack of APOE information, irrelevant outcome, irrelevant study design, or irrelevant publication type (review). The PRISMA flow chart depicting the review process can be seen in Supplementary Figure 1, and the characteristics of included studies can be seen in Supplementary Table 1.

**Figure 1:**
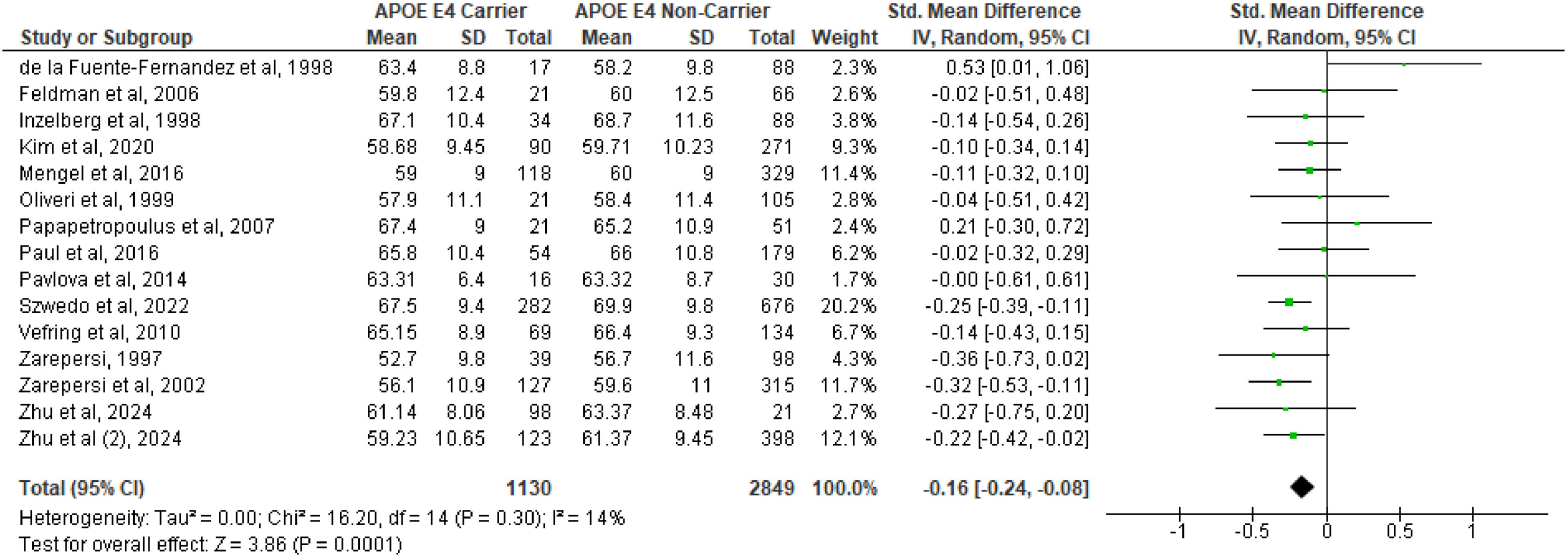
Forest plot for pooled standardized mean difference (SMD) and 95% confidence interval (CI) of Parkinson's disease age of onset between APOE ε4 carriers vs. non-carriers. Analysis was performed using the Random Effects Model. APOE, Apolipoprotein E; and between-study heterogeneity was assessed using the I^2^ statistic.

**Figure 2:**
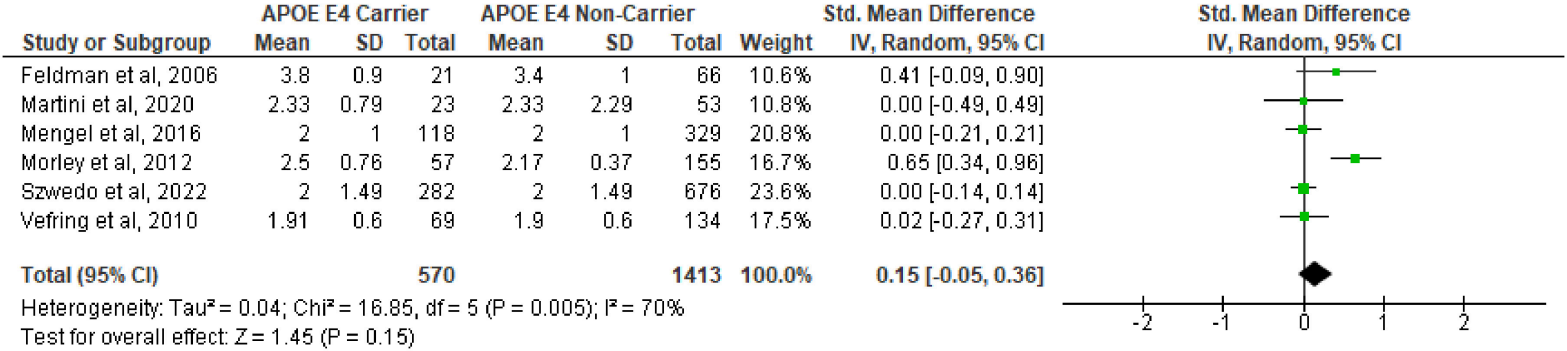
Forest plot for pooled standardized mean difference (SMD) and 95% confidence interval (CI) comparing the Hoehn and Yahr severity scores between Parkinson's disease subjects who are APOE ε4 carriers vs. non-carriers. Analysis was performed using the Random Effects Model. APOE, Apolipoprotein E; and between-study heterogeneity was assessed using the I^2^ statistic.

A total of 14 studies (de la Fuente-Fernández et al., 1998; Feldman et al., 2006; Inzelberg et al., 1998; Kim et al., 2021b; Mengel et al., 2016; Oliveri et al., 1999; Papapetropoulos et al., 2007; Paul et al., 2016; Pavlova et al., 2014; Szwedo et al., 2022; Vefring et al., 2010; Zareparsi et al., 1997, 2002; Zhu et al., 2024) were included in the first pooled analysis on the difference between age of onset between APOE ε4 carriers vs. non-carriers. The analysis showed that APOE ε4 subjects had a significantly earlier age of onset (SMD = −0.16, 95%CI −0.24 to −0.08, *p* = 0.0001, Figure 1), with no substantial heterogeneity (Q = 16.2, df = 14, *p* = 0.30; I^2^ = 14%). However, in the second analysis (Figure 2), which included a total of 6 studies (Feldman et al., 2006; Mengel et al., 2016; Szwedo et al., 2022; Vefring et al., 2010; Martini et al., 2020; Morley et al., 2012), no difference was observed in the severity of PD between APOE ε4 carriers vs. non-carriers, as measured with the Hoehn and Yahr severity scale (SMD = 0.15, 95%CI −0.05 to 0.36, *p* = 0.15) and also based on the MDS-UPDRS I (SMD = 0.02, 95%CI −0.13 to 0.16, *p* = 0.82, Supplementary Figure 2A), MDS-UPDRS II (SMD = 0.14, 95%CI −0.14 to 0.42, *p* = 0.32, Supplementary Figure 2B), and MDS-UPDRS III scales (SMD = 0.03, 95%CI −0.12 to 0.18, *p* = 0.70, Supplementary Figure 2C). There was a significant degree of heterogeneity (Q = 16.85, df = 5, *p* = 0.005; I^2^ = 70%) in the Hoehn and Yahr analysis. Heterogeneity was also observed in the MDS-UPDRS II (Q = 5.98, df = 2, *p* = 0.05; I^2^ = 67%, Supplementary Figure 2B) and MDS-UPDRS III (Q = 18.92, df = 8, *p* = 0.02; I^2^ = 58%, Supplementary Figure 2C), but not in the MDS-UPDRS I analysis (Q = 0.66, df = 2, *p* = 0.72; I^2^ = 0%, Supplementary Figure 2A).

**Figure 3:**
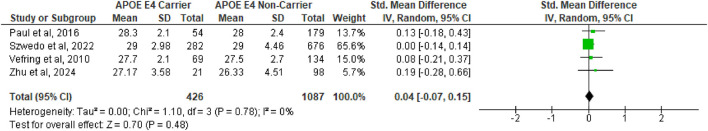
Forest plot for pooled standardized mean difference (SMD) and 95% confidence interval (CI) of Mini Mental State Exam scores between Parkinson's disease patients with APOE ε4 carriers vs. non-carriers status. Analysis was performed using the Random Effects Model. APOE, Apolipoprotein E; and between-study heterogeneity was assessed using the I^2^ statistic.

And finally, in regards to the effect of APOE ε4 carrier status on global cognition, an analysis of 4 included studies (Paul et al., 2016; Szwedo et al., 2022; Vefring et al., 2010; Zhu et al., 2024) revealed no difference in the pooled MMSE scores of subjects (SMD = 0.04, 95%CI −0.07 to 0.15, *p* = 0.48, Figure 3), with no substantial heterogeneity (Q = 1.10, df = 3, *p* = 0.78; I^2^ = 0%).

## Discussion

This study set out to investigate the APOE ε4 status in a group of Indonesia PD patients and their respective families, and elucidate the role of APOE ε4 in PD through a meta-analysis approach. To our knowledge, this is the first study to characterize the APOE gene of Indonesian PD patients, highlighting the allele diversity within the population. Indonesia is a genetically diverse nation, with varying ancestries between the Eastern, Western, and Central islands. Such diversity highlights the importance of exploring genetic associations in population-specific settings. Our findings indicated that the APOE gene distribution aligns with global trends, with a predominance of the APOE ε3 allele. Notably, this study observed a significant prevalence of the ε2 and ε4 alleles, with ε4 found in half of the participants. While high ε4 prevalence was not observed by a previous study on APOE genetics in Indonesia (Hastuti et al., 2015), this study was limited only in one island, and does not account for the other ethnicities.

The relevance of APOE ε4 is emphasized by its links to dementia. In the context of PD, this allele has been associated with an elevated risk of developing dementia (Monsell et al., 2014), and a general decline in cognitive performance (Kim et al., 2021b). Recent studies have suggested that APOE ε4 is linked to faster cognitive decline, with cognitive trajectories correlating with motor deterioration (Jo et al., 2021). The ε4 allele has also been shown as a predictor of cognitive decline in a longitudinal cohort (Umeh et al., 2022).

This study found that individuals carrying the APOE ε4 allele experienced a notably earlier onset of PD compared to non-carriers. The role of APOE ε4 on PD onset has not been explored extensively in the literature, since most studies have focused on its role toward cognitive decline and dementia onset. Earliest reports on the association of APOE e4 carrier status with age of PD onset were those by Zareparsi et al. (1997, 2002) and further supported by Pankratz et al. (2006) wherein subjects with APOE ε4 had a mean PD onset age of 59.7 years which was significantly lower in comparison to subjects homozygous for the more common ε4 allele with a mean onset age of 62.4 years. While many recent studies corroborated these findings (de la Fuente-Fernández et al., 1998; Papapetropoulos et al., 2007), some indicated earlier onset in non-carriers or lacked significant results due to limited statistical power. Thus, the results from our meta-analysis bolsters the concept of an earlier PD onset among APOE ε4 carriers relative to non-carriers.

Several mechanisms have been implicated regarding the potential role of APOE ε4 in earlier PD onset. This gene is known to be involved in neurodegenerative processes and may influence multiple neurodegenerative diseases (Yang et al., 2023). The overlapping effects of APOE on both PD and Alzheimer's Disease (AD), despite their distinct causes, may arise from shared neurodegenerative pathways (Zareparsi et al., 2002). Several studies have shown the role of APOE in α-synuclein pathology, mainly through extracellular signaling pathways that involve APOE (Gallardo et al., 2008).

Interestingly, despite the clear link between APOE ε4 and earlier onset, no significant differences were found in the Mini-Mental State Examination (MMSE) scores between carriers and non-carriers in this study, although these results need to be interpreted with caution due to the small study number and the likelihood of sex-dependent effects that was not analyzed in this study. Additionally, the use of MMSE may be insufficient to detect specific deficits, such as early stage impairments in executive function, visuospatial, and memory domains.

The clear associations between APOE ε4 and AD has led to numerous investigations attempting to examine its potential role as a biomarker of cognitive decline in PD and the occurrence of PDD. Additionally, there are numerous similarities between PD and AD, both in pathological (neuronal loss, protein aggregation) and clinical features (dementia and extrapyramidal symptoms) (Williams-Gray et al., 2009). Many studies have supported the notion, including those that revealed significantly lower cognitive domain scores (Pavlova et al., 2014), or higher odds of subsequent dementia and earlier dementia onset among PD APOE ε4 carriers, and longitudinal studies revealing a more rapid decline in PD APOE ε4 carriers (Morley et al., 2012). There is controversy however, from conflicting studies that show a lack of association between cognitive function in PD with the APOE ε4 status (Mengel et al., 2016), which some may have attributed to sex-specific effects due to findings of steeper cognitive decline in male PD patients carrying the ε4 allele that contrasts the lack of interaction in female subjects (Kim et al., 2021a).

Since this study did not analyze the role of APOE ε2, this limitation can be further explored in the future. A meta-analysis attempting to define the role of APOE genes has shown that while the APOE ε4 allele increases prevalence in AD, it was not associated with sporadic PD prevalence. Instead, the APOE ε2 showed positive associations with sporadic PD prevalence (Huang et al., 2004), although larger susequent genetic studies did not consistently observe similar findings (Federoff et al., 2012). And while most studies on APOE in PD have focused on the disease modifying role of the ε4 allele, there is a possibility of an association of ε2 based meta-analysis studies, although it seems to be more ethnicity dependent (Li et al., 2018). Additionally, this is a preliminary study performed in a limited dataset of PD patients, and future studies in the Indonesian population should aim at exploring a larger population size with the aim of uncovering associations between genes with PD susceptibility, onset age, severity and cognitive decline, through whole genome or whole exome sequencing methods. Due to the highly diverse genetic background across the Indonesian islands, future association studies may potentially uncover clinically relevant genotype-phenotype associations that can impact patient care in the nation. However, most of current efforts in genomic studies within in Indonesia have been small scale studies, and PD patients have been particularly underrepresented, and future collaborations remain key to uncover crucial genetic findings.

## Conclusion

The APOE ε4 allele is common in the Indonesian population and in Indonesian PD subjects in our preliminary clinical study. Based on our meta-analysis, APOE ε4 carriers had a significantly earlier onset relative to non-carriers. No difference was observed in the PD severity and MMSE scores of carriers vs. non-carriers.

##  Publisher's note

All claims expressed in this article are solely those of the authors and do not necessarily represent those of their affiliated organizations, or those of the publisher, the editors and the reviewers. Any product that may be evaluated in this article, or claim that may be made by its manufacturer, is not guaranteed or endorsed by the publisher.

## Data Availability

The original contributions presented in the study are included in the article/Supplementary material, further inquiries can be directed to the corresponding authors.
