# Supplementary material for: Elucidating the role of APOE ε4 gene variants in the clinical manifestation of Parkinson's disease
Source: Front Aging Neurosci. 2025 Oct 17;17:1632480. doi: 10.3389/fnagi.2025.1632480 (PMC12575363; doi:10.3389/fnagi.2025.1632480)
Supplement: Supplementary file 1 [file Data_Sheet_1.docx]

**SUPPLEMENTARY MATERIAL**

**Supplementary Figure 1.** PRISMA Flowchart of the study

**Identification of studies via databases and registers**

Duplicate records removed

(n = 148)

Records identified through electronic database searching

(n = 2203 )

**Identification**

Records screened

(n = 2055)

Records excluded after abstract and title screening

(n = 2003)

**Screening**

Reports excluded after full-text assessment

(n = 34)

Full-text reports assessed for eligibility

(n = 52)

Studies included in review

(n = 18)

**Included**

**Supplementary Figure 2. (A)** Forest plot for pooled standardized mean difference (SMD) and 95% confidence interval (CI) of Parkinson’s Disease MDS-UPDRS I between APOE ε4 carriers versus non-carriers. **(B)** Forest plot for pooled standardized mean difference (SMD) and 95% confidence interval (CI) of Parkinson’s Disease MDS-UPDRS II between APOE ε4 carriers versus non-carriers. **(C)** Forest plot for pooled standardized mean difference (SMD) and 95% confidence interval (CI) of Parkinson’s Disease MDS-UPDRS III between APOE ε4 carriers versus non-carriers. Analysis was performed using the Random Effects Model. APOE = apolipoprotein E, and between-study heterogeneity was assessed using the I² statistic.

**
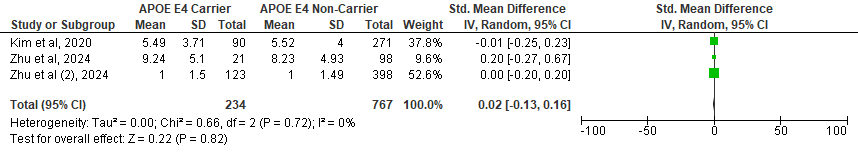

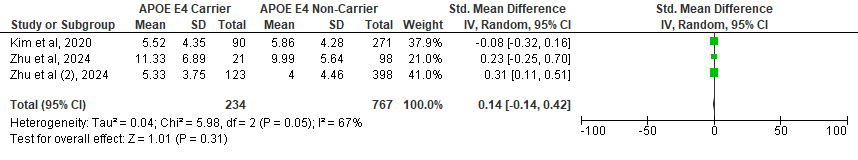

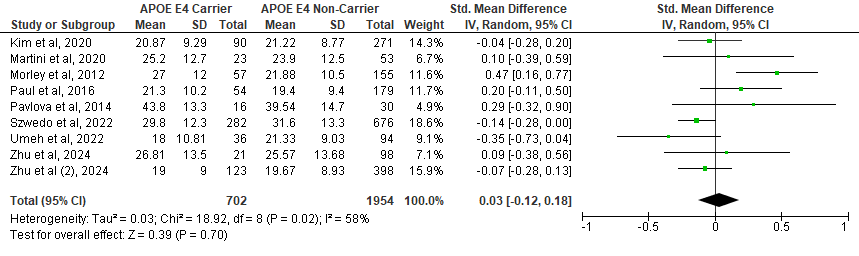
**

**A**

**C**

**B**


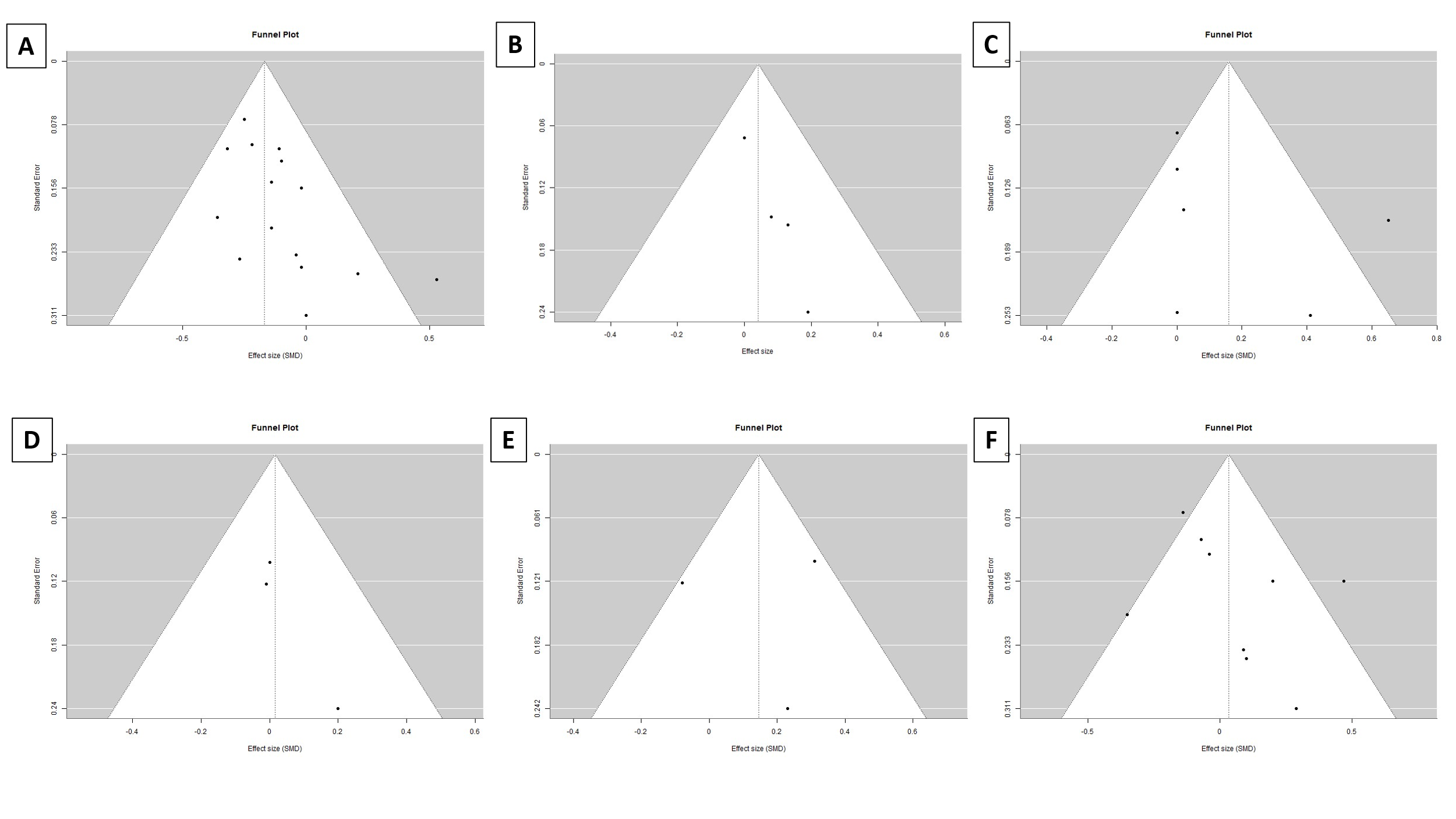
**Supplementary Figure 3.** Funnel plot for (A) age of onset analysis; (B) MMSE scores; (C) Hoehn and Yahr scores; (D) MDS-UPDRS I scores, (E) MDS-UPDRS II scores, and (F) MDS-UPDRS III scores. MMSE = mini-mental state examination, MDS-UPDRS = Movement Disorder Society – Unified Parkinson’s Disease Rating Scal

| **Test of Funnel Plot Assymetry (Egger’s Test) of Studies** | **Z score** | **P value** |
| --- | --- | --- |
| Age of Onset | 2.3785 | 0.0174 |
| MMSE | 1.0341 | 0.3011 |
| Hoehn and Yahr | 0.8113 | 0.4172 |
| MDS-UPDRS I | 0.7697 | 0.4415 |
| MDS-UPDRS II | 0.1119 | 0.9109 |
| MDS-UPDRS III | 0.9769 | 0.3286 |

**Supplementary Table 1.** Characteristics of Included Studies.

| **Number** | **Country, year** | **Authors** | **Total subjects with Parkinson’s Disease** | **APOE E4 carriers** | **APOE E4 non carriers** |
| --- | --- | --- | --- | --- | --- |
| 1 | China | Zhu et al, 2024 | 119 | 98 | 21 |
|  | Parkinson Progression Marker Initiative Dataset | Zhu et al (2), 2024 | 521 | 123 | 398 |
| 2 | Fox Insight Database | Kapan et al, 2023 | 7616 | 1624 | 5992 |
| 3 | Northern Europe | Szwedo et al, 2022 | 958 | 282 | 676 |
| 4 | United States of America | Martini et al, 2020 | 76 | 23 | 53 |
| 5 | United States of America | Papapetropoulus et al, 2007 | 72 | 21 | 51 |
| 6 | United States of America | Morley et al, 2012 | 212 | 57 | 155 |
| 7 | Bulgaria | Pavlova et al, 2014 | 46 | 16 | 30 |
| 8 | South Korea | Kim et al, 2020 | 361 | 90 | 271 |
| 9 | Norway | Vefring et al, 2010 | 203 | 69 | 134 |
| 10 | Israel | Feldman et al, 2006 | 87 | 21 | 66 |
| 11 | Germany | Mengel et al, 2016 | 447 | 118 | 329 |
| 12 | United States of America | Umeh et al, 2022 | 130 | 36 | 94 |
| 13 | United States of America | Paul et al, 2016 | 233 | 54 | 179 |
| 14 | United States of America | Zareparsi et al, 2002 | 442 | 127 | 315 |
| 15 | United States of America | Zareparsi et al, 1997 | 137 | 39 | 98 |
| 16 | Israel | Inzelberg et al, 1998 | 122 | 34 | 88 |
| 17 | Spain | de la Fuente-Fernindez et al, 1998 | 105 | 17 | 88 |
| 18 | Italy | Oliveri et al, 1999 | 126 | 21 | 105 |
